# Surgical Interventions Are Effective for Treating Unruptured Sinus of Valsalva Aneurysms

**DOI:** 10.3389/fcvm.2021.707147

**Published:** 2021-09-06

**Authors:** Juntao Qiu, Enzehua Xie, Yuetang Wang, Wei Wang, Cuntao Yu, Xinjin Luo

**Affiliations:** Department of Cardiovascular Surgery, Fuwai Hospital, National Center for Cardiovascular Diseases, Chinese Academy of Medical Sciences and Peking Union Medical College, Beijing, China

**Keywords:** unruptured sinus of Valsalva aneurysm, surgical repair, right coronary sinus, Dacron patch, cardiovascular lesions

## Abstract

**Background:** This study investigates the optimal management for unruptured sinus of Valsalva aneurysms (USVAs) combined with other cardiovascular lesions.

**Methods:** This retrospective study examined 33 USVA patients who underwent surgical repair from February 1, 2007 to January 31, 2012. We analyzed the surgical procedures and the patients' quality of life after surgery. Additionally, echocardiography follow-up was performed before and after the operation.

**Results:** Most USVAs (87.8%) originated in the right coronary sinus. Aside from one patient who was preoperatively misdiagnosed as having a ruptured sinus of Valsalva aneurysm (SVA). USVAs of the right coronary sinus were addressed by reinforcing this sinus with a Dacron patch through the right ventricle. USVAs were corrected by aortotomy using an autogenous pericardium patch when they originated in the non-coronary or left coronary sinus. Thirty patients (90.9%) were followed up for 22–119 months. No early death, residual fistula or SVA recurrence were found during the follow-up period. They all had a good quality of life and good heart function (New York Heart Association class I–II).

**Conclusions:** Active surgical repair of an USVA can be achieved with satisfactory results in patients combined with other cardiovascular lesions.

## Introduction

Sinus of Valsalva aneurysms (SVAs) are rare cardiac anomaly with an incidence of ~0.09% in bulk autopsy reports (1). SVAs can be congenital or acquired by infection. However, congenital aneurysms are more common. Furthermore, SVAs are more prevalent in the Asian population than in Western populations (2–5). Aneurysm ruptures and hemodynamic changes eventually result in congestive heart failure, which is how SVAs are most commonly diagnosed.

Ruptured SVAs require emergent operation (6). However, whether a USVA needs urgent surgery is controversial. USVAs are usually found when performing other cardiac lesions, and patients are always asymptomatic. Hence, at present, there is no evidence to indicate that surgical intervention is helpful to improve patient outcomes. Moreover, surgery will increase patients' economic burden. To address this issue, we retrospectively analyzed the clinical data for 33 patients who underwent surgery for USVA.

## Methods

This retrospective study analyzed 33 patients with a USVA (27 males, 6 females) who underwent surgery between Feb 1, 2007 and Jan 31, 2012 at Fu Wai Hospital. Their ages ranged from 4.5 to 58 years (mean, 28.5 ± 13.5 years), and their body weights ranged from 17 to 90 kg (mean, 56.0 ± 18.6 kg). SVAs are so rare that we do not see that many cases. To understand the distribution of SVA, we also analyzed 139 patients with ruptured SVAs who underwent surgical repair during the same time period. We hope to describe the clinical and epidemiological characteristics of SVA. All available information regarding the patients was retrieved and analyzed, including diagnoses, presence of additional cardiovascular lesions, operative methodologies, postoperative complications and postoperative echocardiography. The patients were approached by the outpatient department and asked to participate in follow-up telephone calls and questionnaires.

### Statistical Analysis

Kolmogorov–Smirnov tests were performed to detect the normal distribution of continuous variables, which were presented as mean and standard deviation. The categorical variables were described as frequency and percentage (%). Statistical analyses were performed using R version 3.6.3 and Microsoft Excel version 16.16.23.

## Results

### Clinical Results

**Figure 1:**
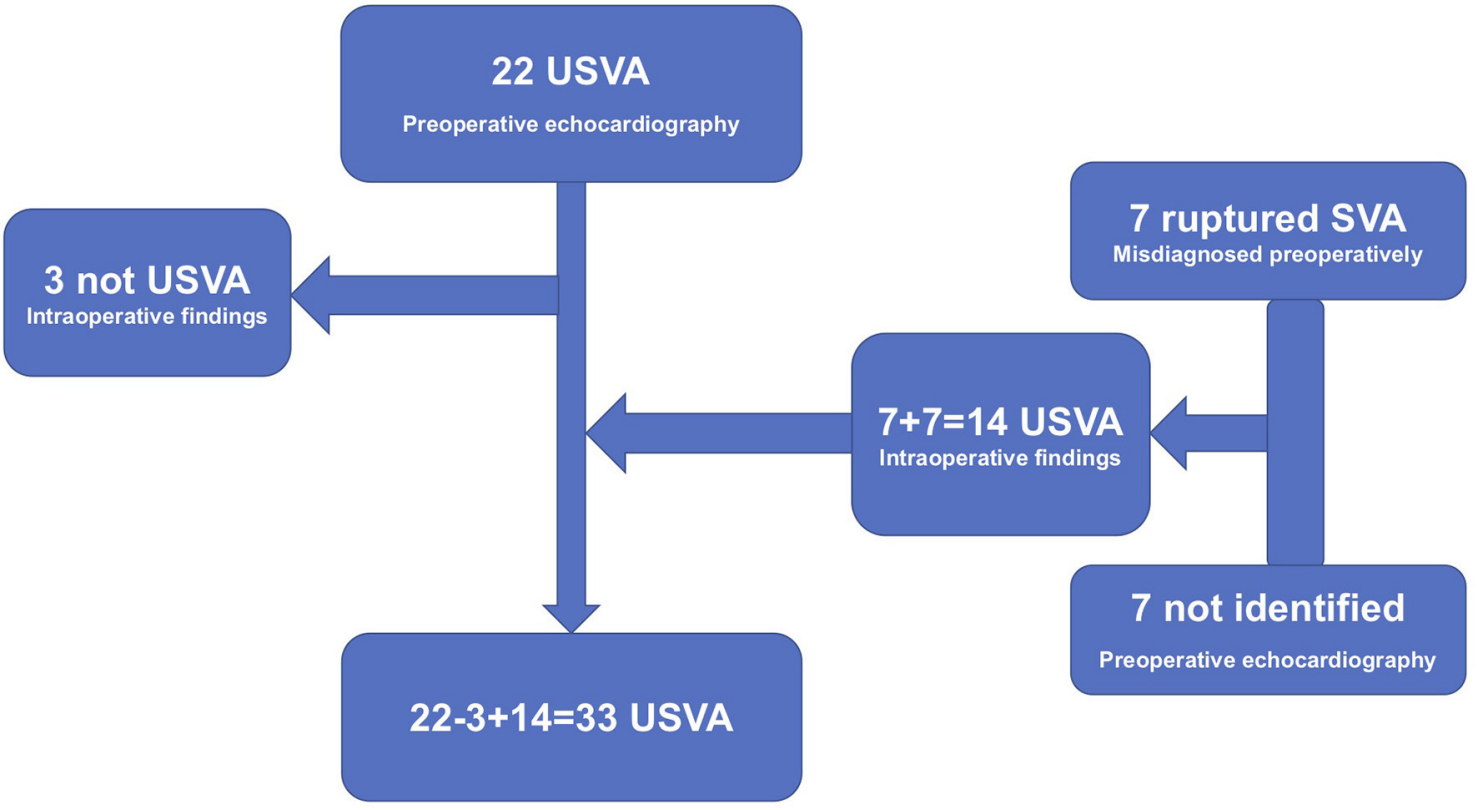
Twenty-two patients were diagnosed with a USVA using preoperative echocardiography. Three patients' diagnoses were revised during the operation. The other 14 USVAs were diagnosed during the operation. Among these 14 patients, seven were misdiagnosed as having a ruptured SVA by preoperatively echocardiography. Another seven patients had lesions that were not identified by preoperatively echocardiography. Thus, the accuracy of preoperative echocardiography was ~52.8% (19/36).

Over the study period (Figure 1), 22 patients were diagnosed with a USVA using preoperative echocardiography. However, during the operation, three patients' diagnoses were revised due to total right coronary sinus expansion without local protrusion of the sinus wall. The other 14 USVAs were diagnosed during the operation. Among these 14 patients, seven were misdiagnosed as having a ruptured SVA (all were indicated as having a right coronary sinus rupture into the right ventricle) by preoperatively echocardiography. These seven patients were diagnosed as ventricular septal defect (VSD). Another seven patients (one with a non-coronary sinus aneurysm and two with a left coronary sinus aneurysm, other four were right coronary sinus aneurysms) had lesions that were not identified by preoperatively echocardiography. Thus, the accuracy of preoperative echocardiography was ~52.8% (19/36). According to Table 1, we can see that most of the USVAs also originated in the right coronary sinus.

**Table 1:** Origin of sinus of Valsalva aneurysms (SVAs) diagnosed at FuWai Hospital from February 1, 2007 to January 31, 2012.

	**Unruptured SVA**	**Ruptured SVA**	**Total**
Right coronary sinus	29 (87.8%)	107 (77.0%)	136 (79.1%)
Non-coronary sinus	2 (6.1%)	30 (21.6%)	32 (18.6%)
Left coronary sinus	2 (6.1%)	2 (1.4%)	4 (2.3%)

*SVA, sinus of Valsalva aneurysm*.

Thirty-three patients were found to have a USVA during the operation, and all of the unruptured sinus of Valsalva aneurysms had been surgically repaired. The surgical procedure was performed through a median sternotomy under complete anesthesia and hypothermic cardiopulmonary bypass. Only one patient was preoperatively misdiagnosed as having a ruptured right coronary sinus aneurysm not associated with any other cardiovascular lesion. The remaining 32 patients presented with a USVA that was associated with other cardiovascular lesions and obtained surgical correction simultaneously (Table 2).

**Table 2:** Cardiac lesions accompanied by an unruptured sinus of Valsalva aneurysm (USVA).

	**Right coronary sinus**	**Non-coronary sinus**	**Left coronary sinus**	**Total**
	**(*n* = 29)**	**(*n* = 2)**	**(*n* = 2)**	
VSD	28 (97%)	1 (50%)	0 (0)	29 (88%)
AI	17 (59%)	1 (50%)	2 (100%)	20 (61%)
Subaortic membrane	3 (10%)	0 (0)	0 (0)	3 (9%)
RVOTS	4 (14%)	0 (0)	0 (0)	4 (12%)
SBE	0 (0)	0 (0)	1 (50%)	1 (3%)
MI/MS	2 (7%)	2 (100%)	1 (50%)	5 (15%)

*VSD, ventricular septal defect; AI, aortic insufficiency; RVOTS, right ventricular outflow stenosis; MI, mitral valve insufficiency; MS, mitral valve stenosis; SBE, sub-acute bacterial endocarditis*.

The surgical interventions that were simultaneously performed, including VSD repair (*n* = 29), aortic valve replacement with a mechanical valve (AVR) (*n* = 5), dredging of the right ventricular outflow tract (*n* = 4), mitral valvuloplasty (*n* = 3), subaortic membrane resection (*n* = 3), mitral and aortic valve replacement with mechanical valves (*n* = 2), aortic valvuloplasty (*n* = 2), and Bentall's operation (*n* = 1).

None of the patients died during surgery. One patient underwent a second AVR 14 days after the first AVR operation due to a perivalvular leak. All other patients were cured and discharged without complication.

### Surgical Methods Employed for USVAs

Three out of the four patients with left coronary sinus (*n* = 2) and non-coronary sinus aneurysms (*n* = 2) underwent a surgical repair for USVA through aortic incision. The aneurysmal neck was repaired with an autogenous pericardial patch in the aortic sinus, and the aneurysmal body was isolated. In the remaining patient, aneurysmal body exclusion was achieved automatically during Bentall's operation.

The various repair techniques and routes employed to treat the remaining 29 patients with USVA of the right coronary sinus are listed in Table 3.

**Table 3:** Repair pathway and surgical procedure for unruptured sinus of Valsalva aneurysms (USVAs) originating from right coronary sinus.

**Repair path for USVAs**	**Unruptured aneurysmal body resected**	**Unruptured aneurysmal body retained**	**Total**
Through the right ventricular chamber (unilateral repair)	3	18	21 (72.4%)
Through aortic incision and the right ventricular chamber (bilateral repair)	3	5	8 (27.6%)
Total	6 (20.7%)	23 (79.3%)	29

*USVA, unruptured sinus of Valsalva aneurysm*.

In the patients in whom a unilateral right ventricular chamber repair path was used, the right coronary sinus was reinforced with a Dacron patch by running suture through the right ventricular chamber. If a VSD was also present (*n* = 20), the same Dacron patch was used to repair the VSD simultaneously. There were 10 patients in whom the aneurysmal body and neck were not repaired, eight in whom the aneurysmal body was preserved but the aneurysmal neck was linear sutured or ligatured, and three in whom both the aneurysmal body and neck were repaired.

Bilateral repair was employed in eight patients. The aneurysmal neck was managed in the right coronary sinus through aortic incision. This was accomplished using an autogenous pericardial patch, a Dacron patch, and a linear suture for repair purposes, respectively. Then, the aneurysmal wall was reinforced with a Dacron patch to repair the VSD through the right ventricular chamber. Among these eight patients, three patients received an aneurismal body resection through the right ventricular chamber, and the incision margin was linearly sutured. However, the isolated aneurysmal body was preserved in the other five patients.

### Follow-Up Results

Thirty patients (90.9%) completed the postoperative follow-up, and the follow-up duration ranged from 22 to 119 months. They all had a good quality of life and good heart function (New York Heart Association class I–II). According to postoperative echocardiography, there were no residual shunts at the sinus aneurysm or VSD repair sites, and there was no sinus aneurysm recurrence.

However, aortic regurgitation proved worthy of attention. Nine patients were preoperatively diagnosed with moderate or severe AI, including three patients who also had aortic stenosis. Eight of them underwent AVR with a mechanical valve. There were no complications during the follow-up period. One patient underwent aortic valve repair due to a valve crevasse detected in the right-non coronary sinus junction during the operation. However, the severity of AI worsened continuously during the follow-up period, and this patient had to undergo a second operation for AVR after 18 months.

The degree of AI for another 24 patients is listed in Table 4, and the intra-operative management for AI and follow-up data for these 24 patients are also shown in Table 4. The data indicate that postoperative AI could be improved using a repair route only through the right ventricle chamber for patients with a USVA originating from the right coronary sinus.

**Table 4:** Conditions of patients with unruptured sinus of Valsalva aneurysms (USVA) accompanied by AI (regurgitation classed as moderate or worse preoperatively).

**Preoperation AI**	**Number (*n*)**	**Intraoperative aortic valve management (*n*)**	**Repair path of sinus aneurysm (*n*)**	**Follow-up period AI**
Mild–Moderate	5	AVP (1)	Bilateral repair (1)	Moderate
		Not handled (4)	Bilateral repair (1)	Mild–Moderate
			Unilateral repair through right ventricle (3)	Trace
Mild	6	Not handled (6)	Bilateral repair (2)	Mild
			Unilateral repair through right ventricle (1)	
			Unilateral repair through right ventricle (3)	Trace
None—Trace	13	Not handled (13)	Bilateral repair (1)	Mild
			Unilateral repair through right ventricle (2)	
			Bilateral repair (3)	None—Trace
			Unilateral repair through right ventricle (6)	
			Unilateral repair through non-coronary sinus (1)	

*AI, aortic insufficiency; AVP, aortic valve plasty*.

## Comment

Congenital SVAs often result in a continuity defect in the aortic wall between the medial aorta and annulus fibrosus. The myxoid and fibrinoid hyaline degeneration can be detected in excised sinus aneurysms and aortic valves by histological examination. The long-term impact of the pulsatile, high-pressure blood flow associated with this type of defective aortic sinus wall results in tissue degeneration and a gradual tumor sample inburst into the adjacent chamber with low pressure, leading to an eventual rupture (7).

When combined with a VSD, the suction caused by high-speed blood flow from a left-to-right shunting further aggravates the protrusion of the local sinus wall, accelerating sinus aneurysm formation. This process also causes prolapse of the aortic valve and introduces AI. In our study, 88% of USVAs and 97% of USVAs from right coronary sinus aneurysms were associated with a VSD. In addition, 61% of USVAs were associated with AI.

Right sinus aneurysms accounted for the majority of USVAs examined. Such a phenomenon is possibly explained by two reasons. Firstly, the wall of right coronary sinus is relatively weak. Secondly, patients combined with ventricular septal defect are more likely to have Valsalva aneurysms. At the location of the ventricular septal defect, the blood flow rate is faster than other locations (Venturi effect), while the fluid pressure is lower (Bernoulli's law). Since the right coronary sinus and ventricular septum are closely located, this might be a possible cause of the dilatation. However, the prevalence of USVAs originating from the left coronary sinus is much higher than that of ruptured SVAs over the same period (6.1–1.4%).

Previous reports, most of which are case reports, have focused on conduction blocks and outflow tract obstructions caused by USVA compression (8–10). There is no unified opinion on whether USVAs require surgical treatment, and many experts believe that observation for USVAs is adequate and that there is no need to intervene when these patients present without symptoms (11–13).

However, we suspected that surgical treatment of USVAs reduces the risk of negative outcomes. In our study, there was one patient who was diagnosed with a “non-coronary sinus aneurysm” by echocardiography in September 2009, with the following description: “Non-coronary sinus local bulge approximately 17 mm × 19 mm, non-coronary leaflet in poor activity, moderate AI and mild mitral valve insufficiency.” At that time, the patient refused treatment because of the lack of symptoms. However, 11 months later, echocardiography indicated that the non-coronary sinus aneurysm had dissected into the anterior leaflet of the mitral valve (A2–A3 area), with an aneurysmal neck thickness of 9 mm; the mitral insufficiency worsened from moderate to severe. The patient finally underwent aneurysm repair accompanied by aortic and mitral valve replacements. The patient might have retained his own mitral valve if surgery had been performed earlier. Therefore, in our study, USVA repair was performed when patients presented with other cardiovascular lesions that needed correction.

It is still unclear whether patients with a USVA that is not associated with other cardiac diseases should be surgically managed. In our study, only one patient misdiagnosed as having a ruptured sinus aneurysm underwent surgical treatment. Therefore, this study does not address this question. Nevertheless, we showed that the accuracy of preoperative echocardiography had for diagnosing USVA was low (precision rate 52.8%). In contrast, the accuracy of preoperative echocardiography for diagnosing ruptured SVA was as high as 99% in our hospital (14). Consequently, more attention should be devoted to suspected cases of USVA, as preoperative misdiagnoses may lead to poor management of these aneurysms.

There are a variety of ways to repair aortic sinus aneurysms, but there is no evidence at present to demonstrate which is the most effective. The findings presented in this study suggest that left coronary and non-coronary sinus aneurysm necks can be repaired using autologous pericardia through aortotomy. In contrast, the methods employed to repair right coronary sinus aneurysms vary considerably. Whether a single right ventricular chamber or bilateral path was utilized, all methods used a Dacron patch to strengthen the sinus wall through the right ventricular chamber. Differences emerge only in terms of aneurysmal body excision and aneurysmal neck repair. One patch could be used to repair both the fistula and associated VSD.

The effectiveness of the various repair techniques is excellent based on postoperative follow-up. To avoid residual shunt, we recommend suturing on normal sinus wall tissue and not using a patch that is too small. Injury to the aortic valve leaflets or coronary ostia should be avoided, and aortic sinus wall deformation should be prevented as much as possible.

During the follow-up period, for the 23 patients with a USVA originating from the right coronary sinus who had no or less-than-moderate AI preoperatively, we found that the postoperative change in the degree of AI was variable based on different surgical interventions. Patients with an operation only through the right ventricular chamber fared better with respect to postoperative AI than patients with an operation through the intra-aortic sinus or patients who underwent aortic valve repair. Five of the eight patients who underwent bilateral repair experienced no difference in the degree of AI compared to their preoperative condition. The condition of the other three patients had worsened AI (one patient experienced moderate AI postoperatively). In contrast, among the 15 patients with an operation through the right ventricular chamber, there were no changes in the degree of AI in seven patients, whereas six patients showed improvement in postoperative AI and two patients had worsened AI (from preoperative trace to postoperative mild AI). However, this worsened AI was considered to have no influence on quality of life. The development of methods by which this disease can be managed is a problem worthy of further research.

Some reports have suggested that patients with a ruptured aortic sinus aneurysm associated with moderate or severe AI should consider an AVR (3, 14). The poor therapeutic performance of valvuloplasty has been attributed to the fact that the aortic valves of these patients are thicker and stiffer due to blood flow aberrations. The poor therapeutic outcomes achieved in patients with unruptured aortic sinus aneurysms in our study support this standpoint. In eight out of nine such cases, AVR was performed immediately during an initial surgical exploration. There was only one patient in whom a second AVR had to be performed due to increased regurgitation 18 months after the first aortic repair.

## Limitations

As a retrospective single-center study, our study also had some limitations. Few cases of left and non-coronary sinus of Valsalva aneurisms are reported. The patients with a USVA that is not associated with other cardiac diseases were lost as follow-up and clinical data were unavailable. Furthermore, we don't have the information among patients who die before hospital admission. We are now enrolling more patients into the registry.

## Conclusion

This study demonstrates the value of surgical intervention for USVA patients, especially patients who also present with other cardiovascular lesions. Thus, we demonstrate that surgical repair of USVAs can be achieved with satisfactory results.

## Data Availability Statement

The raw data supporting the conclusions of this article will be made available by the authors, without undue reservation.

## Ethics Statement

This study was approved by the Ethics Committee of Fuwai Hospital.

## Author Contributions

JQ, XL, and EX developed study design. JQ and EX wrote the paper. JQ, XL, EX, YW, WW, and CY organized patient recruitment. JQ and EX were involved in the statistical analyses and diagramming. All authors contributed to the article and approved the submitted version.

## Conflict of Interest

The research was conducted in the absence of any commercial or financial relationships.

## Publisher's Note

All claims expressed in this article are solely those of the authors and do not necessarily represent those of their affiliated organizations, or those of the publisher, the editors and the reviewers. Any product that may be evaluated in this article, or claim that may be made by its manufacturer, is not guaranteed or endorsed by the publisher.

## Abbreviations

USVAs: unruptured sinus of Valsalva aneurysms
SVA: sinus of Valsalva aneurysms
VSD: ventricular septal defect
AI: aortic insufficiency
RVOTS: right ventricular outflow stenosis
MI: mitral valve insufficiency
MS: mitral valve stenosis
SBE: sub-acute bacterial endocarditis
AVP: aortic valve plasty.
